# Stimulation of constitutive nitric oxide uniquely and compensatorily regulates intestinal epithelial cell brush border membrane Na absorption

**DOI:** 10.14814/phy2.14086

**Published:** 2019-05-10

**Authors:** Balasubramanian Palaniappan, Palanikumar Manoharan, Subha Arthur, Soudamani Singh, Usha Murughiyan, Uma Sundaram

**Affiliations:** ^1^ Department of Clinical and Translational Sciences Joan C Edwards School of Medicine Marshall University Huntington West Virginia

**Keywords:** Constitutive nitric oxide, Na homeostasis, sodium glucose cotransport, sodium proton exchange

## Abstract

In the mammalian small intestine, sodium is primarily absorbed by Na^+^/H^+^ exchange (NHE3) and Na‐glucose cotransport (SGLT1) in the brush border membrane (BBM) of villus cells. However, how enhanced cellular constitutive nitric oxide (cNO) may affect NHE3 and SGLT1 remains unclear. Both in vivo in rabbit intestinal villus cells and in vitro IEC‐18 cells, administration of NO donor, GSNAP, modestly increased cNO. GSNAP stimulated SGLT1 in villus and IEC‐18 cells. The mechanism of stimulation was secondary to an increase in the affinity of SGLT1 for glucose. The change in SGLT1 was not secondary to altered Na‐extruding capacity of the cell since Na^+^/K^+^‐ATPase was decreased by GSNAP treatment. In contrast, GSNAP inhibited NHE3 activity in villus cell BBM. The mechanism of NHE3 inhibition was secondary to reduced BBM transporter numbers. These studies demonstrated that the physiological increase in cNO uniquely regulates mammalian small intestinal NHE3 and SGLT1 to maintain Na homeostasis.

## Introduction

The mammalian small intestine absorbs approximately 7.5 L of water every day, primarily mediated by the absorption of 650 mEq of sodium (Na). The two most important pathways of Na absorption in the small intestine are coupled electroneutral NaCl absorption and nutrient‐dependent Na absorption. Coupled NaCl absorption occurs via the dual operation of Na^+^/H^+^ exchange (NHE3) and Cl^−^/HCO_3_‐exchange (DRA or PAT‐1) located in the brush border membrane (BBM) of absorptive villus, but not secretory crypt cells. Na‐glucose cotransport (SGLT1), also located in the BBM of the villus cells, is the most abundant nutrient‐dependent Na absorptive process in the mammalian small intestine (Hoogerwerf et al. 1996; Sundaram et al. 1997; Donowitz et al. 2005; Coon et al. 2007). SGLT1 is not only important for Na absorption, but also for glucose absorption which is the most abundant nutrient in the diet (Wright et al. 2006). In fact, the foundation of oral rehydration therapy is preserved SGLT1, which is the cornerstone of therapy for diarrheal diseases in developing countries. SGLT1 is a secondary active transport process requiring a favorable transcellular Na gradient which is provided by the Na^+^/K^+^‐ATPase located in the basolateral membrane (BLM) of villus cells (Wright et al. 2007).

Regulation of SGLT1 and NHE3 by various hormones such as aldosterone and immune inflammatory mediators have been demonstrated (Sundaram et al. 1999; De La Horra et al. 2001). Furthermore, in an animal model of chronic intestinal inflammation, resembling human inflammatory bowel disease, it was shown that NHE3 was not affected, while SGLT1 and other Na‐dependent nutrient cotransport processes were inhibited (Sundaram and West 1997; Sundaram et al. 1998a,1998b; Saha et al. 2012).

Nitric oxide (NO) is a highly active molecule shown to regulate multiple gastrointestinal functions. Small amounts of NO (constitutive NO/cNO) regulate normal intestinal physiological processes like motility, blood flow, and mucosal permeability (Shah et al. 2004). In contrast, during intestinal inflammation, high levels of NO (inducible NO/iNO) are produced and is thought to be involved in enterocyte apoptosis and necrosis, intestinal barrier failure resulting in bacterial translocation, reduced colonic motility, secretory diarrhea, and pathogenesis of inflammatory bowel disease (Kubes and McCafferty 2000; Grisham et al. 2002). Intestinal absorption and secretion has been shown to be affected by cNO (Barry et al. 1994; Coon and Sundaram 2003; Coon et al. 2005, 2007, 2008; Arthur et al. 2014). Nevertheless, many NO‐mediated effects on intestinal electrolyte transports are contradictory. For example, in some studies, NO has been shown to enhance electrolyte transport in guinea‐pig small intestine, rat jejunum, ileum, colon, and human colon (MacNaughton 1993; Tamai and Gaginella 1993; Stack et al. 1996; Mourad et al. 2003). However, other studies demonstrated that NO has both proabsorptive and prosecretory roles in cholera toxin‐induced secretion, basal proabsorptive tone in the intestine and reversal of L‐arginine induced fluid secretion (Schirgi‐Degen and Beubler 1998; Turvill et al. 1999; Mourad et al. 2003).

Additionally, previous in vivo and in vitro studies have demonstrated that the inhibition of cNO differentially regulates NHE3 and SGLT1 in intestinal epithelial cells (Coon et al. 2005, 2007, 2008; Palaniappan and Sundaram 2018). In vivo administration of L‐Ng‐Nitroarginine methylester (L‐NAME) to rabbits and in vitro administration to IEC‐18 cells, reduced cNO by the inhibition of cNO synthase (cNOS). Inhibition of cNO stimulated BBM NHE3 in both in vivo and in vitro (Coon et al. 2007). The mechanism of stimulation of NHE3 was secondary to increased BBM transporter numbers. In contrast, diminished cNO resulted in the inhibition of SGLT1 in rabbit villus cells and IEC‐18 cells (Coon et al. 2005, 2008). Kinetic and molecular studies demonstrated that the mechanism of inhibition was secondary to a reduction in the affinity of SGLT1 for glucose. The inhibition of SGLT1 was not secondary to the reduction in the Na‐extruding capacity of cells, since Na^+^/K^+^‐ATPase was unchanged and the observed effects persisted in BBMV where this is not an issue. In summary, these studies demonstrated that in vivo and in vitro inhibition of cNO stimulated BBM NHE3, but inhibited SGLT1. Thus, inhibition of cNO differentially regulates NHE3 and SGLT1 in the mammalian small intestine. However, the effect of physiological increases in cNO on these two primary Na absorptive pathways in the mammalian small intestine remains unknown. Thus, it is important to determine the effect of moderate, direct increases in cNO on SGLT1 and NHE3 to fully elucidate the mechanism of physiological changes in cNO on these two important BBM Na absorptive processes.

## Methods

### Ethical approval

New Zealand White male rabbits (2.5–2.7 kg) were obtained from Charles River Laboratories International, Inc., USA. All animal handling, treatments and euthanization were carried out according to the protocol approved by Marshall University institutional animal care and use committee (IACUC) regulations. Before and throughout the experiments, animals had free access of water and food and were kept in 12 h light–dark cycle room in the animal facility.

### Drug treatment

To induce nitric oxide production in the rabbits, 1 mL of 500 nmol/L N‐(*β*‐D‐glucopyranosyl)‐N2‐acetyl‐S‐nitroso‐D, L‐penicillaminamide (GSNAP; Calbiochem, USA), a conventional NO donor, was injected intramuscularly. Control animals were injected with sterile distilled water. The animals were euthanised with 120 mg/kg of pentobarbital sodium (approved by IACUC) through the ear vein after 24 h of GSNAP treatment.

### Villus cell isolation and BBM vesicle preparation

Villus cells were isolated from the GSNAP treated and control rabbit intestine by a calcium chelation technique as previously described (Sundaram et al. 1991, 1997). BBM vesicles (BBMV) from rabbit intestinal villus cells were prepared using divalent cation (Mg^2+^) chelation and differential centrifugation technique as previously reported (Sundaram et al. 1997, 1998a). BBMV were suspended in an appropriate reaction medium for each uptake experiment or with an appropriate buffer for molecular studies.

### Cell culture and treatment

Rat IEC‐18 cells were grown in DMEM supplemented with 0.2 U/mL of insulin, 0.5 mmol/L *β*‐hydroxybutyrate and 10% fetal calf serum, and incubated at 37°C with 10% CO_2_ in a humidified atmosphere. Cells grown as postconfluent monolayers were used for all the experiments. The cells were treated with 250 nmol/L GSNAP (Calbiochem, USA) for 24 h to increase cNO or with vehicle in control cells prior to the uptake experiments.

### Measurement of NO

Total NO levels were measured in cells (villus and IEC‐18) by Griess reaction colorimetric assay (Cayman chemicals, USA) according to the manufacturer's instruction. Briefly, GSNAP treated and untreated villus and IEC‐18 cells were lysed with phosphate‐buffered saline (PBS) by sonication. The cell lysate was then centrifuged and the clear supernatants were used for the assay to measure total NO (nitrite+nitrate). Samples were read at 540 nm and total NO concentrations were determined with the nitrate standard curve.

### NHE3 uptake studies in IEC‐18 cells

Uptake studies for Na^+^/H^+^ exchange were performed in confluent IEC‐18 cells as described previously (Palaniappan and Sundaram 2018). Briefly, the cells were incubated for 10 min in buffer containing 70 mmol/L TMACl, 50 mmol/L NH_4_Cl, 5 mmol/L KCl, 1 mmol/L MgSO_4_, 2 mmol/L CaCl_2_, 5 mmol/L glucose, 15 mmol/L Tris‐HEPES (pH 7.4) and washed with wash buffer containing 120 mmol/L TMACl and 15 mmol/L Tris‐HEPES (pH 7.4). Uptake was initiated by incubating the cells for 2 min with a reaction medium containing Na‐free buffer with 10 *μ*Ci of ^22^Na and 1 mmol/L NaCl in the presence or absence of 50 *μ*mol/L EIPA (NHE3 inhibitor). The reaction was stopped and the cells were washed twice with ice‐cold phosphate‐buffered saline. The cells were then incubated with 1 N NaOH for 20 min at 70°C, to digest the cells, before addition of 4 mL of scintillation fluid (Ecoscint; National Diagnostics). Radioactivity was determined in a Beckman 6500 Beta scintillation counter.

### Na‐glucose cotransport uptake studies in IEC‐18 cells

Na‐glucose cotransport uptakes were performed in postconfluent IEC‐18 cells using a previously described protocol (Palaniappan and Sundaram 2018). Briefly, the cells were washed and incubated with Na‐free buffer containing 130 mmol/L TMACl, 4.7 mmol/L KCl, 1 mmol/L MgSO_4_, 1.25 mmol/L CaCl_2_, 20 mmol/L HEPES (pH 7.4 at 37°C) for 10 min. Uptake was initiated by incubating the cells for 2 min in a Tris‐HEPES (pH 7.4) reaction medium containing 130 mmol/L NaCl, 10 *μ*Ci of ^3^H‐*O*‐methyl glucose (3‐OMG) and 100 *μ*mol/L 3‐OMG in the presence or absence of 1 mmol/L phlorizin (SGLT1‐inhibitor) and 10 mmol/L glucose. The reaction was stopped and the cells were washed twice with ice‐cold Na‐free buffer containing 25 mmol/L D‐glucose. The cells were then processed as described above. SGLT1‐specific uptake was calculated by subtracting uptake with and without phlorizin.

### Uptake studies in rabbit villus cell BBMV

Na^+^/H^+^ exchange uptake was measured in BBMV by rapid‐filtration technique as previously described (Manokas et al. 2000; Coon and Sundaram 2003; Coon et al. 2005). Briefly, 5 *μ*L of BBMV was suspended in 300 mmol/L mannitol, 50 mmol/L Tris‐MES (pH 5.5) or 50 mmol/L Tris‐HEPES (pH 7.5), and incubated in 95 *μ*L of reaction medium containing 300 mmol/L mannitol, 50 mmol/L Tris‐HEPES (pH 7.5 at room temperature), 1 mmol/L ^22^NaCl and with or without 1 mmol/L amiloride. At 60 sec, uptake was arrested by mixing with ice‐cold stop solution (300 mmol/L mannitol, 50 mmol/L Tris‐HEPES (pH 7.5)). The results were expressed as Na^+^/H^+^ exchange uptake in picomoles per milligram protein at 60 sec. To derive kinetic parameters of BBM Na^+^/H^+^ exchange, the numbers obtained from kinetic experiments were analyzed using GraphPad Prism 7 (San Diego, CA).

For Na‐glucose cotransport uptake studies, BBMV uptake studies were performed by the rapid‐filtration technique as previously described (Sundaram et al. 1997, 1998a; Turner and Black 2001). In brief, 5 *μ*L of BBMV was suspended in Na‐free buffer containing 130 mmol/L TMACl, 4.7 mmol/L KCl, 1 mmol/L MgSO_4_, 1.25 mmol/L CaCl_2_, 20 mmol/L HEPES (pH 7.4). The BBMV was then incubated in 95 *μ*L of reaction medium that contained 130 mmol/L NaCl, 4.7 mmol/L KCl, 1 mmol/L MgSO_4_, 1.25 mmol/L CaCl_2_, 20 mmol/L HEPES (pH 7.4 at room temperature), 10 *μ*Ci of ^3^H‐O‐methyl glucose (OMG), and 100 *μ*mol/L OMG in the presence or absence of 1 mmol/L phlorizin. At 90 sec, uptake was arrested by mixing with ice‐cold stop solution (Na‐free buffer) containing 25 mmol/L D‐glucose. The uptake experiment results were expressed as Na‐glucose cotransport uptake in nanomoles per milligram protein at 90 sec. To determine the kinetic parameters of BBM Na‐glucose cotransport, the numbers obtained with kinetics experiments were analyzed using GraphPad Prism 7 (San Diego, CA).

### Enzyme measurement

Na^+^/K^+^‐ATPase was measured as *P*_*i*_ liberated in GSNAP treated and untreated villus cells and IEC‐18 cellular homogenates, as previously described (Forbush 1983; Palaniappan and Sundaram 2018). Enzyme‐specific activity was expressed as nanomoles of *P*_*i*_ released per milligram protein per minute.

### Western blot analyses

Western blot analyses of villus cell and IEC‐18 cell BBM were performed as described previously (Palaniappan and Sundaram 2018). BBM solubilized in RIPA buffer (50 mmol/L Tris HCl pH 7.4, 1% Igepal, 150 mmol/L NaCl, 1 mmol/L EDTA, 1 mmol/L PMSF, 1 mmol/L Na_3_VO_4_, 1 mmol/L NaF) containing protease inhibitor cocktail (SAFC Biosciences) was mixed with sample buffer (100 mmol/L Tris, 25% glycerol, 2% SDS, 0.01% bromophenol blue, 10% 2‐ME, pH 6.8) and separated on a custom made 8% poly acrylamide gel. The separated proteins were transferred to BioTrace PVDF membrane and after blocked probed with anti‐NHE3 antibodies and anti‐SGLT1 antibodies raised in chicken (Invitrogen custom antibody services, USA) and anti‐ Ezrin antibodies (ab231907, Abcam, USA) raised in rabbit, at dilution of 1:1000 overnight at 4°C in fat‐free milk containing 1× TBS‐Tween 20. Horseradish peroxidase coupled rabbit antichicken antibody (Prod # 31401, Invitrogen, USA) for NHE3 and SGLT1, goat antirabbit antibody (sc‐2357, Santa Cruz, USA) for Ezrin at dilution of 1:10,000 for 1 h at room temperature in fat‐free milk containing 1× TBS‐Tween 20 were used to detect the binding of specific primary antibodies of both transporters. The resulting chemiluminescence with ECL Detection Reagent (GE Healthcare) was measured by autoradiography. NHE3 and SGLT1 protein density was quantitated via a densitometric scanner FluorChem™ instrument (Alpha Innotech, San Leandro, CA).

### Protein quantification

For all the uptake and molecular studies, proteins were quantified with the DC™ protein assay kit (Lowry's method) according to manufacturer's protocols (Bio‐Rad).

### Statistical analysis

Results presented represent means ± SE of experiments performed and calculated by the GraphPad Prism 7 (San Diego, CA). All uptakes were done in triplicate. Student's *t*‐test was performed for statistical analysis.

## Results

### Effect of GSNAP on intracellular NO levels

**Figure 1:**
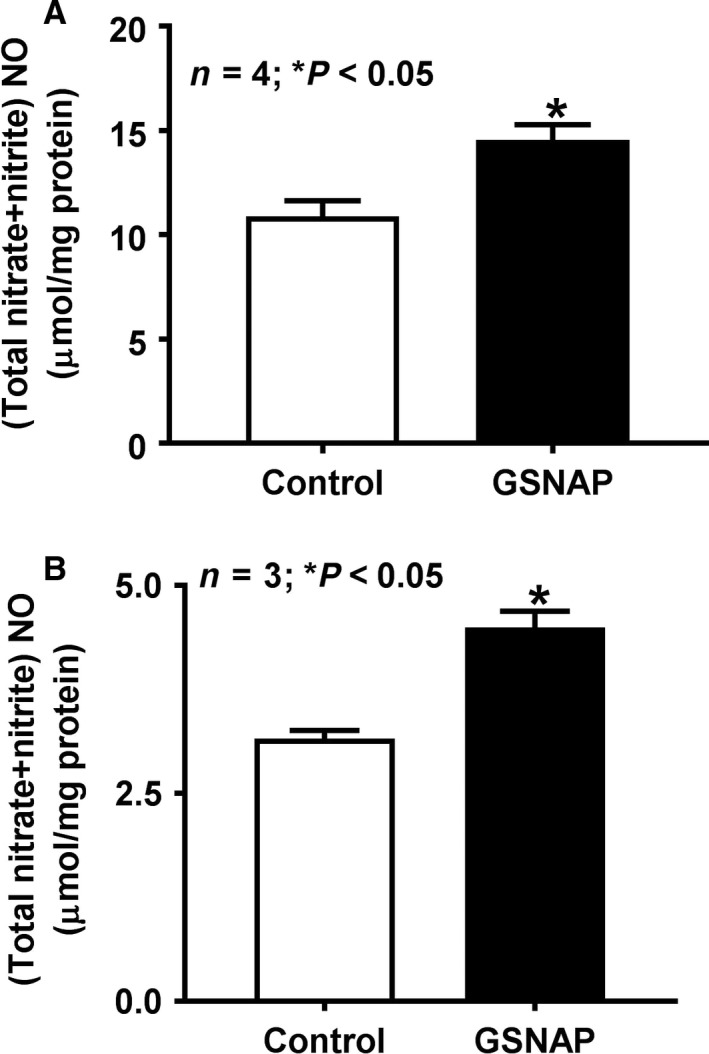
Effect of GSNAP on intracellular NO levels. (A) In vivo treatment with NO donor GSNAP in rabbits modestly, but significantly increased cNO levels in villus cells compared to control. (B) In vitro treatment of IEC‐18 cells with GSNAP also increased intracellular cNO. Thus, in vivo or in vitro, GSNAP increased intracellular cNO in intestinal epithelial cells.

The lowest possible dose of GSNAP that produced a reproducible increase in cNO was used in all experiments. In vivo treatment of rabbits with 500 nmol/L GSNAP modestly but significantly increased the intracellular cNO levels (Fig. 1a). Therefore, this concentration of GSNAP was used in all in vivo experiments. Similarly, when IEC‐18 cells were treated with 250 nmol/L GSNAP, it increased the intracellular cNO to modest levels as well (Fig. 1b). This concentration of GSNAP was then used in all subsequent in vitro studies.

### Effect of GSNAP on Na^+^/H^+^ exchange in villus BBMV in vivo

**Figure 2:**
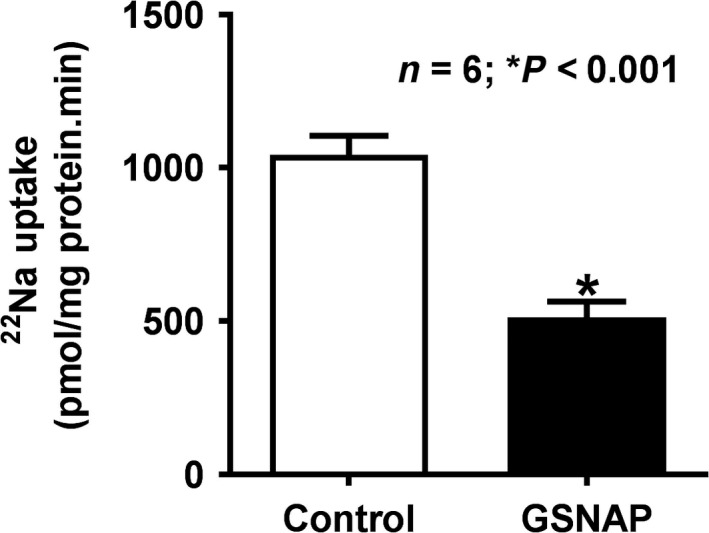
Effect of GSNAP on Na^+^/H^+^ exchange in villus BBMV. In GSNAP‐treated rabbit villus BBMV, proton gradient‐dependent, amiloride‐sensitive Na^+^/H^+^ exchange was significantly diminished when compared with control villus BBMV. Thus, Na^+^/H^+^ exchange inhibition is due to increased cNO levels in rabbit villus cells.

In in vivo, GSNAP inhibited amiloride‐sensitive ^22^Na uptake in villus cell BBMV prepared from control and GSNAP‐treated rabbits (Fig. 2; control 1043 ± 62 pmol/mg protein·min and GSNAP 512.3 ± 51.4; *n* = 6, *P* < 0.001). These data demonstrated that the stimulation of cNO production decreased Na^+^/H^+^ exchange in the villus cell BBMV.

### Effect of GSNAP on Na^+^/H^+^ exchange in IEC‐18 cells

**Figure 3:**
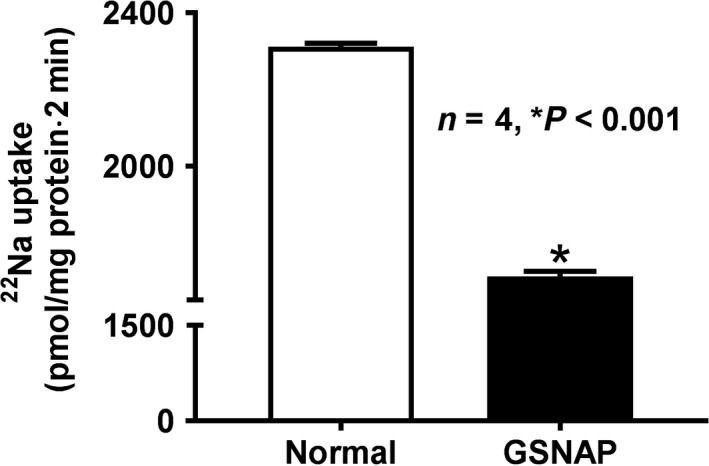
Effect of GSNAP on Na^+^/H^+^ exchange in IEC‐18 cells**.** Direct in vitro stimulation of cNO with GSNAP in IEC‐18 significantly diminished proton gradient‐dependent, EIPA‐sensitive Na uptake when compared with control IEC‐18 cells. These data indicated that an increased NO inhibited NHE3 in IEC‐18 cells

Na^+^/H^+^ exchange was significantly diminished in GSNAP‐treated IEC‐18 cells compared to control as shown in Figure 3 (1711 ± 13.4 pmol/mg protein·2 min in GSNAP‐treated IEC‐18 cells and 2312 ± 89 in control; *n* = 4, *P* < 0.001). These data demonstrated that the stimulation of cNO production decreases BBM Na^+^/H^+^ exchange in vitro in IEC‐18 cells.

### Effect of GSNAP on Na‐glucose cotransport in villus cell BBMV in vivo

**Figure 4:**
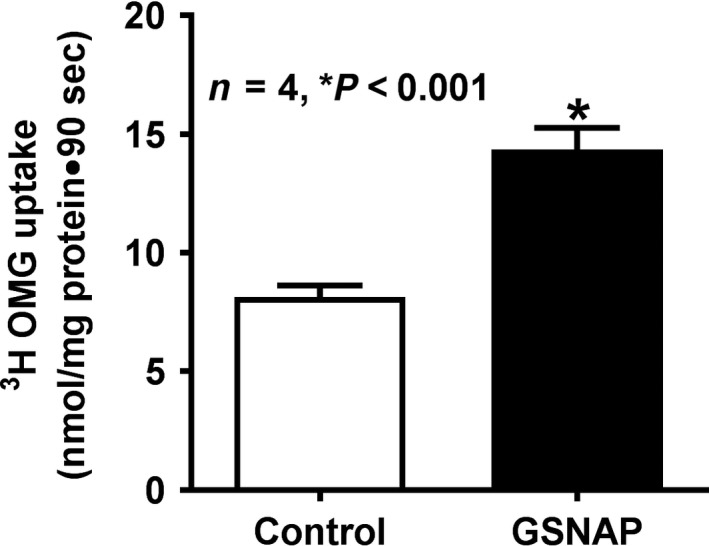
Effect of GSNAP on Na‐glucose cotransport in villus cell BBMV. In GSNAP‐treated rabbit villus BBMV, Na‐dependent glucose cotransport (^3^H‐OMG uptake) was significantly increased when compared with control. Thus, Na‐glucose cotransport stimulation is due to increased cNO levels in rabbit villus cells.

In in vivo, GSNAP treatment stimulated Na‐dependent phlorizin‐sensitive ^3^H‐OMG uptake in villus cell BBMV (Fig. 4; control 8.1 ± 0.5 nmol/mg protein·90 sec and GSNAP 14.4 ± 0.9; *n* = 4, *P* < 0.001). These data demonstrated that the stimulation of cNO production increases Na‐glucose cotransport in the villus cell BBMV.

### Effect of GSNAP on Na‐glucose cotransport in IEC‐18 cells

**Figure 5:**
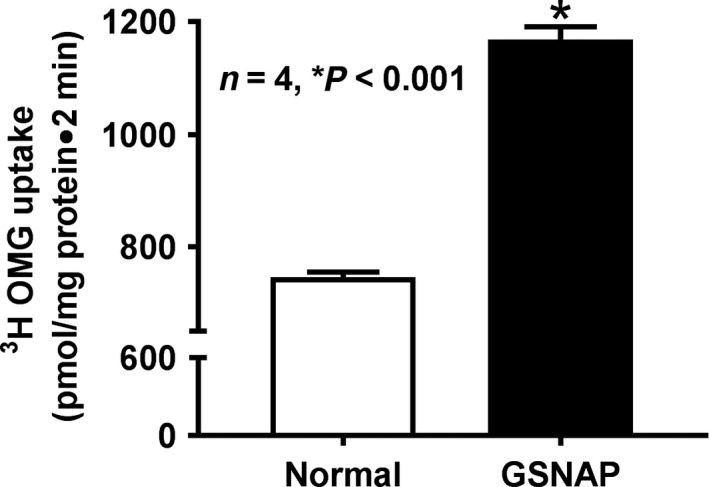
Effect of GSNAP on Na‐glucose cotransport in IEC‐18 cells. Direct in vitro stimulation of cNO with GSNAP in IEC‐18 significantly increased Na‐dependent and phlorizin‐sensitive ^3^H‐OMG uptake when compared with control IEC‐18 cells. These data also indicated that intracellular increase in NO stimulated SGLT1 activity in IEC‐18 cells.

Na‐glucose cotransport was significantly increased in GSNAP‐treated IEC‐18 cells compared to control (Fig. 5; 1168 ± 23.2 pmol/mg protein·2 min in GSNAP‐treated cells and 745.9 ± 9.8 in control; *n* = 4, *P* < 0.001). These data demonstrated that the stimulation of cNO production increases BBM Na‐glucose cotransport in vitro in IEC‐18 cells.

### Effect of GSNAP on Na^+^/K^+^‐ATPase activity

Since Na^+^/K^+^‐ATPase in the basolateral membrane (BLM) provides the favorable Na gradient for SGLT1 in intact cells, its activity was determined in cellular homogenates from all experimental conditions. GSNAP treatment decreased Na^+^/K^+^‐ATPase activity in IEC‐18 cells (24.2 ± 1.0 nmol *P*_*i*_/mg protein·min in control and GSNAP 15.3 ± 1.5; *n* = 4, *P* < 0.01) and in villus cells (control 14.8 ± 0.7 nmol *P*_*i*_/mg protein•min and GSNAP 11.6 ± 0.5; *n* = 6, *P* < 0.05). This indicated that the effect of GSNAP on Na‐glucose cotransport was not secondary to enhanced transcellular Na gradient.

### Kinetic studies of NHE3 inhibition in rabbit villus cell BBMV and IEC‐18 cells

**Figure 6:**
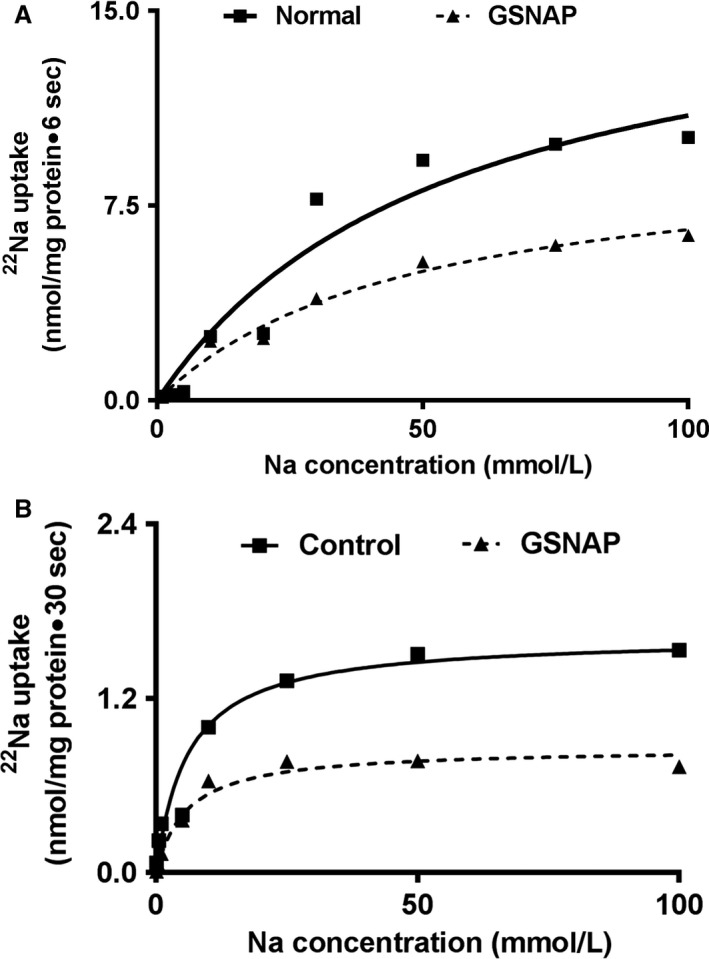
Kinetic studies of NHE3 inhibition in rabbit villus cell BBMV and IEC‐18 cells. (A) In rabbit villus cell BBMV, as the extra vesicular Na concentration was increased, Na uptake was stimulated and then reached steady state. The kinetic parameters demonstrated that the affinity [1/Michaelis constant (1/*K*_m_)] for ^22^Na uptake was not altered by the increase in cNO production (Table 1A). However, the maximal rate of uptake (*V*_max_) was significantly inhibited by GSNAP treatment (Table 1A). (B) Similarly in IEC‐18 cells, as the extracellular Na concentration was increased, Na uptake was stimulated and subsequently became saturated in all conditions. The kinetic parameters demonstrated that the affinity for ^22^Na uptake was not altered by the increase in cNO production in IEC‐18 cells (Table 1B). However, the maximal rate of uptake (*V*_max_) was significantly inhibited by GSNAP treatment in these cells (Table 1B).

Kinetic studies were performed to determine the mechanism of inhibition of Na^+^/H^+^ exchange in the small intestine due to increased cNO. Uptake for all the concentrations was carried out at 6 sec since the initial rate of uptake for Na^+^/H^+^ exchange in BBMV was linear for at least 10 sec. As the concentration of extra vesicular Na was increased, the uptake of ^22^Na was stimulated and subsequently became saturated in all conditions (Fig. 6A). Table 1 shows the kinetic parameters derived from these experiments. The kinetic parameters demonstrated that the affinity [1/Michaelis constant (1/*K*_m_)] for ^22^Na uptake was not altered by the increase in cNO production (Table 1A). However, the maximal rate of uptake (*V*_max_) was significantly inhibited by GSNAP treatment (Table 1A). These data demonstrated that an increase in cNO production inhibits Na^+^/H^+^ exchange in rabbit villus cells secondary to a decrease in the number of BBM exchangers rather than altered affinity of the exchangers for Na (Fig. 6A).

**Table 1:** Kinetic parameters of Na+/H+ exchange in rabbit villus BBMV and IEC‐18 cells. (A) Rabbit villus cell BBMV: The maximal rate of uptake of sodium (*V*_max_) was significantly decreased in villus BBMV from GSNAP‐treated rabbits; however, the affinity (1/*K*_m_) for sodium uptake was unaltered; (B) IEC‐18 cells: The maximal rate of uptake of Na was also significantly decreased in GSNAP‐treated IEC‐18 cells. However, the affinity of the exchanger for Na was unchanged between control and GSNAP‐treated IEC‐18 cells

(A) NHE3 Kinetics – Villus cell BBMV		
	*V*_max_ (nmol/mg protein 6·sec)	*K*_m_ (mmol/L)
Control	17.0 ± 2.2	55.3 ± 0.7
GSNAP	9.7 ± 1.7*	47.6 ± 7.2

(B) NHE3 Kinetics – IEC‐18 cells		
	*V*_max_ (nmol/mg protein 30·sec)	*K*_m_ (mmol/L)
Control	1.6 ± 0.1	5.9 ± 0.7
GSNAP	0.8 ± 0.1*	4.9 ± 0.5

* *P* < 0.01, *n* = 3.

In IEC‐18 cells, uptake for all the concentrations was carried out at 30 sec because the initial rate of uptake for Na^+^/H^+^ exchange in IEC‐18 cells was linear for at least 60 sec. As the concentration of extracellular Na was increased the uptake of ^22^Na was stimulated and subsequently became saturated in all conditions (Fig. 6B). The *K*_m_ for Na was not altered in GSNAP‐treated IEC‐18 cells (Table 1B). However, the *V*_max_ was significantly decreased in IEC‐18 cells treated with GSNAP (Table 1B). These data indicated that the mechanism of NHE3 inhibition by GSNAP in IEC‐18 cells is also secondary to a decrease in the number of exchangers rather than a decrease in the affinity of the exchangers for Na.

### Kinetic studies of SGLT1 stimulation in rabbit villus cell BBMV and IEC‐18 cells

**Figure 7:**
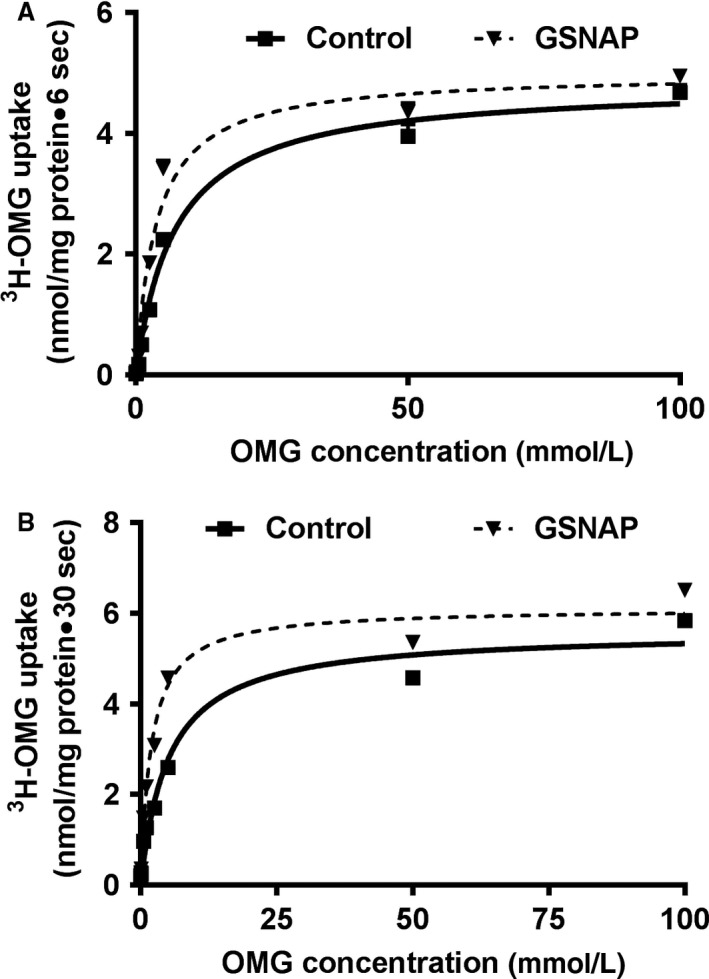
Kinetic studies of SGLT1 stimulation in rabbit villus cell BBMV and IEC‐18 cells. (A) In rabbit villus cell BBMV, as the extravesicular glucose concentration was increased, Na‐dependent glucose uptake was stimulated and then reached steady state. The kinetic parameters demonstrated that the affinity for ^3^H‐OMG uptake was significantly stimulated by the increase in cNO (Table 2A). However, the maximal rate of uptake was not altered by GSNAP treatment (Table 2A). (B) Comparably in IEC‐18 cells, as the extracellular glucose concentration was increased, glucose uptake was stimulated and subsequently became saturated in all conditions. The kinetic parameters demonstrated that the affinity for ^3^H‐OMG uptake was significantly stimulated by the increase in cNO in IEC‐18 cells as well (Table 2B). However, the maximal rate of uptake was not altered by GSNAP treatment in these cells (Table 2B).

Kinetic studies were performed to determine the mechanism of stimulation of Na‐glucose cotransport in the small intestine due to increased cNO. Uptake for all the concentrations was carried out at 6 sec since the initial rate of uptake for Na‐dependent glucose in BBMV was linear for at least 10 sec. As the concentration of extra vesicular OMG was increased, the uptake of Na‐dependent ^3^H‐OMG uptake was stimulated and subsequently became saturated in all conditions (Fig. 7A). Table 2A shows the kinetic parameters derived from these studies. The kinetic parameters demonstrated that the affinity for glucose was significantly stimulated by increased cNO. However, the *V*_max_ was not altered by GSNAP treatment (Table 2A). Thus, these data demonstrated that an increase in cNO production stimulated Na‐glucose cotransport in rabbit villus cells secondary to an increase in the affinity of the cotransporter for glucose rather than an increase in the number of cotransporters (Fig. 7A).

**Table 2:** Kinetic parameters of Na‐glucose co‐transport in rabbit villus BBMV and IEC‐18 cells. (A) Rabbit villus cell BBMV: The maximal rate of uptake of glucose was not affected in villus BBMV from GSNAP‐treated rabbits; however, the affinity for glucose uptake was significantly increased in GSNAP‐treated rabbits. (B) IEC‐18 cells: The affinity for glucose was also significantly increased in GSNAP‐treated IEC‐18 cells. However, the maximal rate of uptake of glucose was unchanged between control and GSNAP‐treated IEC‐18 cells

(A) SGLT1 Kinetics – Villus cell BBMV		
	*V*_max_ (nmol/mg protein·6 sec)	*K*_m_ (mmol/L)
Control	4.8 ± 0.1	7.2 ± 0.6
GSNAP	5.0 ± 0.1	3.8 ± 0.3*

(B) SGLT1 kinetics – IEC‐18 cells		
	*V*_max_ (nmol/mg protein·30 sec)	*K*_m_ (mmol/L)
Control	5.6 ± 0.3	5.2 ± 0.2
GSNAP	6.1 ± 0.1	1.9 ± 0.1*

* *P* < 0.01; *n* = 6.

* *P* < 0.01; *n* = 4.

In IEC‐18 cells, uptake for all the concentrations was carried out at 30 sec because the initial rate of uptake for Na‐dependent glucose cotransport in IEC‐18 cells was linear for at least 60 sec. As the concentration of extravesicular OMG was increased, the uptake of Na‐dependent ^3^H‐OMG was stimulated and subsequently became saturated in all conditions (Fig. 7B). The kinetic parameters demonstrated that the affinity for ^3^H‐OMG uptake was significantly stimulated by increased cNO (Table 2B). However, *V*_max_ was not altered by GSNAP treatment (Table 2B). Thus, these data demonstrated that an increase in cNO production also stimulated Na‐glucose cotransport in IEC‐18 cells secondary to an increase in the affinity of the cotransporters for glucose rather than an increase in the number of BBM cotransporters.

### NHE3 molecular studies

**Figure 8:**
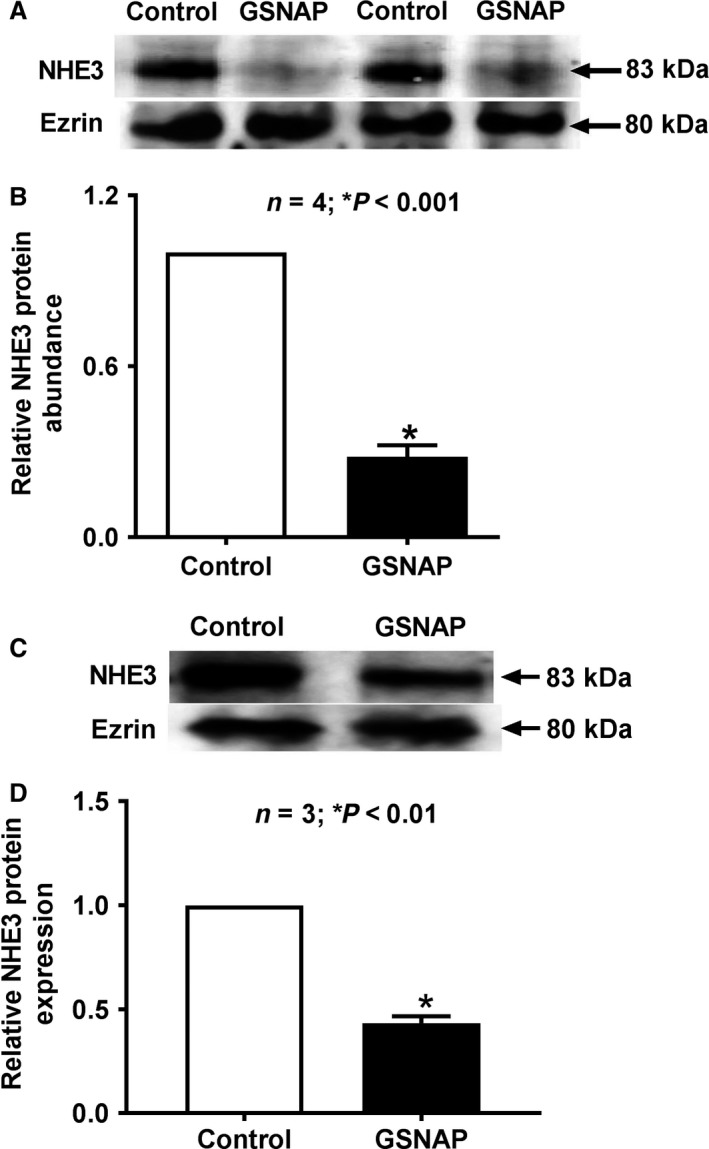
NHE3 molecular studies – effect of GSNAP on villus cell BBM NHE3 protein expression. (A) Representative Western blot shows that in GSNAP‐treated BBM of rabbit villus cells there is reduction of NHE3 protein expression compared with control villus cell BBM. (B) Densitometry quantitation confirmed that treatment with GSNAP significantly decreased BBM NHE3 levels in villus cells. (C) Similarly in IEC‐18 cells, increased cNO significantly downregulated BBM NHE3 protein expression compared with control IEC‐18 cells. (D) Densitometry quantitation confirmed that treatment with GSNAP significantly decreased BBM NHE3 levels in IEC‐18 cells as well.

Na^+^/H^+^ exchange is mediated by NHE3 in the BBM of intestinal epithelial cells. Western blot analysis showed that the immunoreactive NHE3 protein levels in the villus cell BBM were significantly decreased in animals treated with GSNAP (Fig. 8A). Densitometric quantitation shown in Figure 8B, confirmed these findings. Immunoreactive NHE3 protein levels were also decreased in the BBM of IEC‐18 cells by GSNAP treatment (Fig. 8C). Densitometric quantitation shown in Figure 8D, confirmed these findings. The decrease in NHE3 protein in conjunction with kinetic studies demonstrated that the mechanism of inhibition of NHE3 is secondary to a decrease in the number of BBM exchangers rather than a decrease in the affinity of the exchangers for Na.

### SGLT1 molecular studies

**Figure 9:**
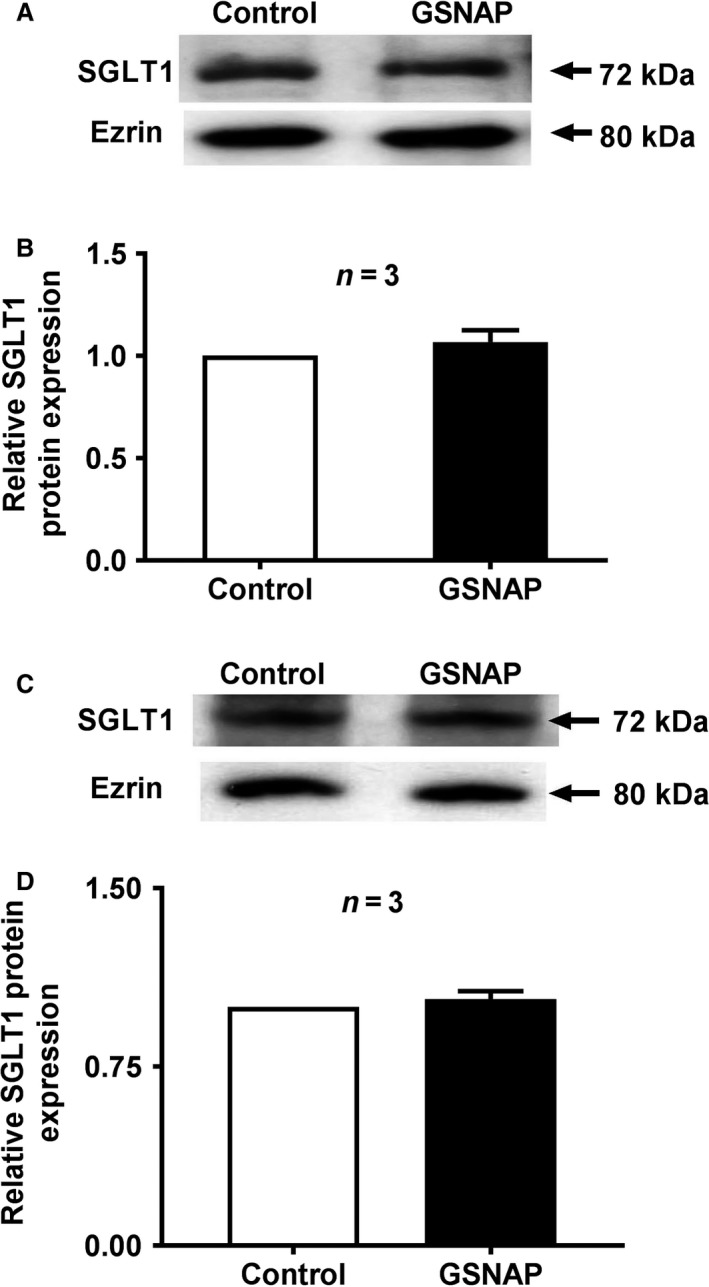
SGLT1 molecular studies – effect of GSNAP on villus cell BBM SGLT1 protein expression. (A) Representative Western blot shows that in GSNAP‐treated BBM of rabbit villus cells there is no change in SGLT1 protein expression compared with control villus cell BBM. (B) Densitometry quantitation confirmed that treatment with GSNAP did not affect BBM SGLT1 levels in villus cells. (C) Similarly in IEC‐18 cells, increased cNO did not affect BBM SGLT1 protein expression compared with control BBM of IEC‐18 cells. (D) Densitometry quantitation confirmed that treatment with GSNAP did not alter BBM SGLT1 levels in IEC‐18 cells as well.

As shown in Figure 9A, Western blot analyses demonstrated that GSNAP treatment did not affect SGLT1 immunoreactive protein levels in rabbit villus cell BBM (Fig. 9B). Western blot analysis also showed that GSNAP treatment did not affect SGLT1 immunoreactive protein levels in IEC‐18 cell BBM (Fig. 9C). Densitometric quantitation shown in Figure 9D confirmed the findings. In conjunction with kinetic studies, these molecular studies demonstrated that the mechanism of stimulation of SGLT1 is secondary to an increase in the affinity of the cotransporter for glucose rather than an increase in the number of cotransporters in the BBM.

## Discussion

This study demonstrates that a moderate increase in cNO stimulated SGLT1 in vivo in rabbit intestinal villus cells and in vitro in IEC‐18 cells. In contrast, an elevation in cNO inhibited NHE3 in vivo in rabbit intestinal villus cells and in vitro in IEC‐18 cells. The mechanism of stimulation of SGLT1 was secondary to enhanced affinity of the cotransporter for glucose without a change in the number of BBM cotransporters. Whereas, the mechanism of inhibition of NHE3 was secondary to an increase in the number of exchangers in the BBM without a change in the affinity of the exchanger for Na. Thus, it is likely that cNO uniquely and possibly compensatorily regulates the primary Na absorptive pathways in the mammalian small intestine to maintain cellular Na homeostasis.

This study illustrates that moderate increase in cNO in vivo increases intestinal glucose absorption by stimulating SGLT1 in villus cells. These findings are diametrically opposite to those seen when cNO production was reduced in vivo with L‐NAME in rabbits. This resulted in inhibition of SGLT1 secondary to diminished affinity of the cotransporter for glucose without a change in the number of BBM cotransporters (Coon et al. 2005). The kinetic parameters are comparable for control villus cells in both studies, with increased affinity when cNO is increased as shown in this study, while *K*_m_ was decreased when cNO was inhibited as previously shown (Coon et al. 2005). Thus, an increase in intracellular cNO stimulates SGLT1 while a decrease in cNO inhibits SGLT1 in mammalian intestinal villus cells.

In vivo cNO inhibition may have multiple other effects, which may in turn affect SGLT1 activity. Therefore, in vitro studies in IEC‐18 cells were performed to determine cNO increase mediated specific alterations in SGLT1 activity. Enhanced intracellular cNO also increased SGLT1 activity in IEC‐18 cells. The mechanism of stimulation of SGLT1 was secondary to enhanced affinity of the cotransporter for glucose without a change in the maximal rate of uptake of glucose. Consistent with this, molecular studies did not show any change in the BBM immunoreactive levels of SGLT1. These findings were identical to the in vivo observations in rabbits when cNO was increased. However, these findings are completely opposite to those demonstrated when cNO production was directly reduced in vitro in IEC‐18 cells. Direct inhibition of cNO in IEC‐18 cells resulted in inhibition of SGLT1 secondary to a decrease in the affinity of the cotransporter for glucose without a change in the number of BBM cotransporters (Coon et al. 2008; Palaniappan and Sundaram 2018). In IEC‐18 cells while the *K*_m_ for control in the current and prior studies were comparable, it was shown to be substantially decreased in the prior study when cNO was diminished (Coon et al. 2008) and in this study *K*_m_ was significantly increased when cNO is enhanced. Thus, whether in vivo or in vitro*,* in two different species, when cNO is increased or decreased it subsequently stimulated or inhibited SGLT1, respectively, by exactly the same mechanism, specifically by altering the affinity of the cotransporter for glucose. As mentioned before, the primary glucose absorption in mammalian intestine is via SGLT1. Therefore, alteration of SGLT1 activity by cNO levels could be central to the altered glucose homeostasis and pathophysiology of diabetes, which affects almost ten percent of the American population (Selvin and Ali 2017).

A recent study showed that NO is a significant player in the pathology of gestational diabetes (Usman et al. 2018). Another study has established that systemic glucose metabolism is modulated through enteric nitric oxide synthase (Abot et al. 2018). Moreover, NO was found to be the regulator of glucose utilization in gut‐brain axis (Fournel et al. 2017). In this context, this study establishes that NO through the regulation of SGLT1 in the intestine may modulate glucose homeostasis and therefore may be involved in the pathology of diabetes.

Whether in vivo or in vitro, the stimulation of SGLT1 is not secondary to altered Na‐extruding capacity of the cell although Na^+^/K^+^‐ATPase was decreased. Since the mechanism of stimulation of SGLT1 was secondary to enhanced affinity of the cotransporter for glucose, cNO appears to modulate SGLT1 at the posttranslational level by affecting the affinity of the cotransporter for glucose. Altered affinity may be secondary to altered phosphorylation and or glycosylation of the cotransporter. In a prior study, it was demonstrated that when cNO production was inhibited, it reduced intracellular cGMP, and via protein kinase G increased the glycosylation of SGLT1 which resulted in the inhibition of its activity (Arthur et al. 2014). The intracellular pathway responsible for the increase in affinity resulting in the stimulation of SGLT1 by increased cNO is yet to be determined.

Apart from altering glucose homeostasis, stimulation of SGLT1 by enhanced cNO will also alter Na homeostasis. Perhaps to compensate for this intracellular Na imbalance, in cNO stimulated cells, the other primary Na absorptive pathway, namely NHE3, appears to be inhibited. This observation is pertinent to conditions associated with deregulated Na absorption such as hypertension. Based on most recent guidelines, 46% of U.S. adults, about 103 million, have hypertension (Fryar et al. 2017; Whelton et al. 2018). Numerous randomized trials and observational studies have demonstrated a direct relationship between assimilation of dietary sodium and blood pressure (Aburto et al. 2013; He et al. 2013). Furthermore, there is a consistent effect of lowering sodium assimilation on blood pressure among those with hypertension (Whelton et al. 1998; Sacks et al. 2001). A recent Cochrane meta‐analysis of data from 35 trials (He et al. 2013) found that a 100 mmol reduction in 24‐h urinary sodium led to a significant reduction in systolic/diastolic blood pressure of 5.4/2.8 mm Hg among hypertensive individuals. Finally, studies have demonstrated an increased risk of mortality for high‐sodium assimilation and a direct relationship with total mortality, even at the lowest levels of sodium intake (Cook et al. 2016).

In vivo stimulation of intracellular cNO inhibits NHE3 activity in villus cells. The mechanism of inhibition is secondary to a decrease in the number of exchangers in the BBM of villus cells without an alteration in the affinity of the exchanger for Na. These findings are also diametrically opposite to those demonstrated when cNO production was reduced in vivo in rabbits. This resulted in the stimulation of villus cell NHE3 activity. The mechanism of stimulation of NHE3 was secondary to enhanced transporter numbers in the BBM without a change in the affinity of the exchanger for Na (Coon et al. 2007). The kinetic parameters are comparable for control villus cells in both studies, with diminished *V*_max_ when cNO is increased as shown in this study, whereas *V*_max_ was increased when cNO was decreased as previously shown (Coon et al. 2007).Finally, possible mechanisms of stimulation of NHE3 include increased de novo synthesis of protein, enhanced trafficking to BBM and/or enhanced mRNA stability of NHE3 transcription. Thus, unlike SGLT1, an increase in intracellular cNO inhibits NHE3 while a decrease in cNO stimulates NHE3 in mammalian intestinal villus cells.

To avoid untoward complications of in vivo increases in cNO, in this study in vitro direct stimulation of intracellular cNO at physiological levels was done which produced results opposite that noted above. Specifically, increased cNO inhibited NHE3 in IEC‐18 cells. The mechanism of inhibition was secondary to decreased BBM transporter numbers. This effect was also seen in human colon cancer Caco2 cells (Gill et al. 2002). In contrast, when cNO was directly inhibited in IEC‐18 cells, it stimulated NHE3 in these cells by increasing the BBM exchanger numbers. Kinetic studies in prior and current study are consistent with this. In IEC‐18 cells while the *V*_max_ for control in the current and prior studies were comparable, it was shown to be substantially increased in the prior study when cNO was diminished and in this study *V*_max_ was significantly decreased when cNO is enhanced (Coon et al. 2008). Thus, when cNO is increased or decreased, it subsequently inhibits NHE3 or stimulates NHE3 by exactly the same mechanism, by altering BBM transporter numbers.

In contrast to SGLT1, the mechanism of inhibition of villus cell NHE3 by increased cNO is secondary to a decrease in exchanger numbers. The decrease in BBM NHE3 may be at the transcriptional level. Transcriptional regulation of NHE3 may occur at three levels: (1) diminished de novo NHE3 mRNA synthesis, (2) reduced NHE3 mRNA stability and/or (3) NHE3 promoter regulation; the sequence of rat NHE3 core promoter region between nucleotides −118 to +59 contains the essential transcription factor interacting sites of Sp 1 family members as well as AP1/CREB (Kiela et al. 2006). Alternatively, since a percentage of NHE3 is in the cytosol at baseline, altered cytosol to membrane trafficking may be the cause of enhanced transporter numbers in the BBM.

The concept of the two primary Na absorptive pathways in the intestine being compensatorily regulated to maintain Na homeostasis by cNO is consistent with the observation that NHE3 and SGLT1 do indeed regulate one another. When NHE3 was silenced in IEC‐18 monolayers with NHE3 siRNA, the cells demonstrated decreased NHE3 activity, mRNA and protein. However, in NHE3 silenced cells, SGLT1 activity, mRNA, and protein in the BBM were significantly increased. Thus, inhibition of NHE3 expression compensatorily increased the expression and function of SGLT1 and vice versa in the BBM of intestinal epithelial cells. Thus, this study demonstrate that the major Na absorptive pathways function compensatorily to regulate Na absorption in intestinal epithelial cells.

This study, for the first time, demonstrates that an increase in intracellular cNO that is not pathological, compensatorily stimulates SGLT1 while inhibiting NHE3 in intestinal epithelial cells from rabbits and rats, in vivo and in vitro*,* respectively. Therefore, cNO directly, broadly and uniquely regulates intestinal epithelial cell BBM Na absorption by compensatorily regulating NHE3 and SGLT1.

## Conflict of Interest

No conflicts of interest, financial or otherwise, are declared by the authors.
